# Noisy and Restless Insanity

**Published:** 1863-08

**Authors:** K. McLeod


Noisy and Restless Insanity.—*Hydrocyanic Acid*—A case is related in which the patient was noisy and restless, incessantly in motion, constantly shouting and screaming, and for long periods sleepless. She was completely delirious, and had not during the day a moment's repose. Four minims of dilute hydrodyanic acid were given every hour, in half an ounce of camphor mixture. Immediately after the first dose she became quiet and still, and slept at night. She continued better until the medicine was discontinued, when she at once relapsed to her old state. This may prove a valuable hint in some of these distressing and unmanageable cases.—(Dr. K. McLeod,)—*Id.*

